# Carcinoma of the Stomach

**Published:** 1868-03-01

**Authors:** J. W. Dora

**Affiliations:** Mattoon, Ill.


### CARCINOMA OF THE STOMACH.

BY J. W. DORA, M.D., MATTOON, ILL.

I HAVE recently had the opportunity of performing a *post-mortem* examination (in company with Drs. Morris, Bridges, and Willcox), upon a case of carcinoma of the stomach, and it may be of some interest to the profession to learn something of the history of the symptoms, and of the autopsical developments, as such cases are comparatively rare in common practice, and very difficult to diagnose, on account of the obscurity of the symptoms in most cases (and, by the way, correct diagnosis is of more importance than any thing else in cancerous affections generally), as the treatment is utterly unavailing, and the prognosis universally unfavorable, in carcinoma of the stomach especially.

The subject of carcinomatous affection of the stomach was Philip Crow, age 51 years, native of Ohio, farmer and trader, of robust, healthy constitution, bilious lymphatic temperament; weight, ordinarily, 175 pounds; height, five feet nine inches; habits of life active. He first began to complain of gastric irritation early in September last, but had felt some dyspeptic symptoms for some three years previous, but paid no attention to it, attributing it to his irregular habits in eating, as he was almost constantly traveling by railroad back and forth to New York with stock, and eating hasty railroad meals, and losing sleep. During one of his visits East, in early autumn, he was attacked with pain in the stomach, nausea, and vomiting, which continued for several days, and was treated by some physician in the city of New York for gastralgia, and received temporary benefit. But not wishing to give a lengthy detail of the progress of the case, I will simply state that I learned from the patient, during my first visit to him, on the evening of November the 22nd, that from the time that the gastric disturbance began in September, he had constantly experienced a gnawing pain in his stomach, when empty, and a dull, heavy, aching pain after meals even of the lightest character, and, also, a very noticeable but marked feature in the case was a continuous pain, extending from the stomach to the left side of the chest and shoulder, so much so that the left arm became very lame. This symptom induced me, at first, to diagnose the case as neuralgia, and prescribed accordingly. There was also a peculiar capriciousness of the appetite, even while the patient was able to attend to business, and travel about, the peculiarity of which was a constant tendency to change diet, not relishing any dish more than two or three times in succession. The symptoms continued without any marked change, except the continued decline and emaciation. The tongue heavily coated with a yellow coat, and bowels obstinately constipated all the time; pulse ranging from 80 to 90; skin cool; no headache during any of the time, and free from pain in any part of his system, except the stomach and left chest. He was visited alternately by Drs. Morris, Bridges, Willcox, and myself, during the two months that he survived after I first saw him, and was treated for the first two or three weeks rather empirically, without success, we merely treated symptoms; but about the expiration of a month, the emaciation enabled us to discover very distinctly that there was a tumor in the epigastrium, well marked, hard, and resisting to the touch, with considerable tenderness, and an undue prominence of the liver, and those of us who were visiting the case at that time pronounced it to be carcinoma of the stomach, with adhesions to the liver and anterior wall of the epigastric region of the abdominal cavity, consequently our treatment became merely palliative. The diet had been, principally, warm sweet milk, four to eight ounces, about five or six times daily, which sustained the vital forces remarkably for a time. Our prognosis, as given to the patient and friends, of course, was unfavorable; whereupon the patient, like all dying men, was anxious to have more council called in, and we suggested several eminent physicians of Chicago; viz., Dr. N. S. Davis, Dr. J. A. Allen, and Dr. Johnson. The two former not being able to make the visit, Prof. Johnson came down, and examined the patient, and corroborated our previous diagnosis and prognosis of the case. The patient very patiently awaited his final summons to the untried world, which transpired on the 19th inst., from inanition. *Post mortem* was made some ten hours after death: Rigor mortis well marked, emaciation very great. The contents of the abdomen only were examined. Upon opening the abdominal cavity, we found that the omentum as such entirely minus, what there was left of it was agglutinated, in puckered folds, to the transverse colon, and there were extensive adhesions existing between the colon and the scirrhus mass which had once been the stomach, and also extensive adhesions to the liver, spleen, and parietes of the abdomen, both anteriorly and posteriorly, and to the diaphragm. The pancreas was entirely obliterated, and converted into a nodulated mass, adhering to the posterior surface of the stomach. After removing the stomach with considerable difficulty, we could not have recognized it as said organ, had it not been that we were in search of it (*i. e.*), from any resemblance which it retained of that organ. The entire tissue of the stomach was one scirrhus mass, involving all its coats on the posterior surface. It was nodulated and perforated in several places, yet the orifices were comparatively healthy. Neither the cardiac nor the pyloric were obstructed or constricted in appearance, that we could detect, but the caliber was very much lessened, especially in its longitudinal aspect; in fact the entire organ was contracted to about one-half its normal dimensions. And there was one other peculiarity discovered; viz., there was upon the entire track of the small intestines, as well as throughout the foliæ of the mesentery, small tuberculous-looking bodies, as large as a split pea, of a yellow color, which, I believe, have been named by some writers embryo cancers, or scirrhus lymphatic glands. The diaphragm and peritoneal lining of upper part of abdominal cavity were also thickly studded with these cancerous growths, if such they were. We were not permitted to remove any portion of the diseased tissue for microscopical examination, as we should like to have done, as it had been requested by the patient that no portion of his tissue should be removed from the body. It is remarkable that any human being could have survived so long, with such an entire arrest of nutrition, and such extensive lesions of the digestive apparatus, as existed in this case. But it may be attributed, in part, to the principle of vital tenacity, which we find very strongly marked in some cases, together with the small amount of nutrition derived from the milk diet, taken during the last two months of the patient's illness.

Mr. Editor please excuse the length to which I have detailed this report.

